# Characterization of a Conserved Interaction between DNA Glycosylase and ParA in *Mycobacterium smegmatis* and *M. tuberculosis*

**DOI:** 10.1371/journal.pone.0038276

**Published:** 2012-06-04

**Authors:** Feng Huang, Zheng-Guo He

**Affiliations:** National Key Laboratory of Agricultural Microbiology, Center for Proteomics Research, College of Life Science and Technology, Huazhong Agricultural University, Wuhan, China; University of Padova, Medical School, Italy

## Abstract

The chromosome partitioning proteins, ParAB, ensure accurate segregation of genetic materials into daughter cells and most bacterial species contain their homologs. However, little is known about the regulation of ParAB proteins. In this study, we found that 3-methyladenine DNA glycosylase I MsTAG(Ms5082) regulates bacterial growth and cell morphology by directly interacting with MsParA (Ms6939) and inhibiting its ATPase activity in *Mycobacterium smegmatis*. Using bacterial two-hybrid and pull-down techniques in combination with co-immunoprecipitation assays, we show that MsTAG physically interacts with MsParA both *in vitro* and *in vivo*. Expression of MsTAG under conditions of DNA damage induction exhibited similar inhibition of growth as the deletion of the *parA* gene in *M. smegmatis*. Further, the effect of MsTAG on mycobacterial growth was found to be independent of its DNA glycosylase activity, and to result instead from direct inhibition of the ATPase activity of MsParA. Co-expression of these two proteins could counteract the growth defect phenotypes observed in strains overexpressing MsTAG alone in response to DNA damage induction. Based on protein co-expression and fluorescent co-localization assays, MsParA and MsTAG were further found to co-localize in mycobacterial cells. In addition, the interaction between the DNA glycosylase and ParA, and the regulation of ParA by the glycosylase were conserved in *M. tuberculosis* and *M. smegmatis*. Our findings provide important new insights into the regulatory mechanism of cell growth and division in mycobacteria.

## Introduction

A typical feature of *Mycobacterium tuberculosis*, the causative agent of tuberculosis, is that it can maintain a non-replicating state for long periods of time in a hostile host-cell environment [1], [2]. However, little is known about the underlying mechanism involved in regulation of chromosome segregation and cell growth in *M. tuberculosis* and its related mycobacterial species. *Mycobacterium smegmatis* is a relatively fast-growing and non-pathogenic mycobacterium species and has been widely used as a model organism to study the gene regulatory mechanisms in mycobacteria [3].

Most bacterial chromosomes encode ParAB proteins or their homologs which play essential roles in ensuring accurate segregation of genetic materials [4]. Generally, ParA and ParB are encoded by the same operon in the chromosome and usually act in collaboration [5]. ParA homologs (such as Soj in *Bacillus subtilis*), which are Walker A cytoskeletal ATPases [6], are responsible for the rapid movement of bacterial chromosomal origin regions (oriC) towards cell poles [5], [7]–[9]. Interestingly, Soj was also shown to play an important role in the regulation of DNA replication initiation and control of sporulation [10]–[12]. ParB has been shown to form higher-order nucleoprotein complexes at partitioning sites (parS) near oriC that are required for efficient chromosomal segregation [13]–[15]. Interestingly, the ATPase activity of ParA has been shown to be required for its function in bacterial chromosome partitioning [16], [17]. Biochemical and structural analysis of *Thermus thermophilus* Soj/ParA showed that a mutant form of the protein deficient in ATP binding lost its DNA binding ability [18]. ATP binding with Soj promotes focus formation and is required for septal localization in *B. subtilis*. However, the SojK16A mutant, which lacks ATP binding activity, localizes throughout the cytoplasm [10].

Both *M. tuberculosis* and *M. smegmatis* genomes were recently found to contain parS sequences and *parAB* genes encoding homologs of ParA and ParB segregation proteins [4], [19]. Library screening through transposon mutagenesis suggested that *parAB* genes are indispensable for *M. tuberculosis* H37Rv [20]. ParA (encoded by Ms6939) of *M. smegmatis* (GenBank accession number CP000480) was found to directly interact with ParB (encoded by Ms6938) and enhance its affinity for origin-proximal parS sequences *in vitro* [4], [21]. Antisense expression of *parA* hinders the growth of *M. smegmatis* [22], while overexpression of MsParA causes the cells to become filamentous and multinucleoidal, indicating defects in cell-cycle progression [21]. Therefore, a tight regulation of ParA activity is critical for normal chromosome segregation and cell cycle progression in mycobacteria. However, the mechanism of ParA regulation and the proteins involved remain to be characterized.

3-methyladenine DNA glycosylases remove 3-methyladenine from alkylated DNA and are widely found in prokaryotic and eukaryotic organisms, including *M. tuberculosis* and *M. smegmatis* [19], [23]–[26]. However, besides their known function as a DNA glycosylase involved in DNA damage and repair, little is known about their other possible functions. In this study, mycobacterial 3-methyladenine DNA glycosylases (TAG) have been linked to the regulation of ParA function and bacterial growth for the first time. We uncovered a novel mechanism of regulation of mycobacterial cell growth and division in which TAG directly interacts with ParA and inhibits its ATPase activity. Furthermore, the interaction between the DNA glycosylase and ParA and the regulation of the latter by the former were shown to be conserved in both *M. tuberculosis* and *M. smegmatis*. Our findings provide important new insights into the regulatory mechanism of cell growth and division in mycobacteria.

## Materials and Methods

### Bacterial Strains, Plasmids, Enzymes and Chemicals

The host strain *Escherichia coli* BL21 (Novagen) and pET28a vector (Novagen) were used to express the *M. smegmatis* [3] proteins. The plasmids pBT, pTRG and *E. coli* XR reporter strains for the bacterial two-hybrid assays were purchased from Stratagene. pGEX-4T-1 were purchased from Pharmacia. Restriction enzymes, T4 DNA ligase, DNA polymerase, modification enzymes, deoxynucleoside triphosphates (dNTPs) and all antibiotics were purchased from TaKaRa Biotech. Polymerase Chain Reaction (PCR) primers were synthesized by Invitrogen (Suppl Table S1). All plasmids constructed in this study are listed in Suppl Table S2. Ni-NTA (Ni^2+^nitrilotriacetate) agarose was obtained from Qiagen.

### Cloning, Expression and Purification of Recombinant Proteins

*parA* and *Tag* genes from *M. smegmatis* or *M. tuberculosis* genome were amplified using their PCR primers (Suppl Table S1) and cloned into the prokaryotic expression vector pET28a or pGEX-4T-1. *E. coli* BL21 was used to express the recombinant proteins [27]. The recombinant *E. coli* BL21 cells were grown in a 1 L LB medium up to an OD_600_ of 0.6. Protein expression was induced by the addition of 1 mM isopropyl b-D-1-thiogalactopyranoside (IPTG) at 16°C for 18 h. The harvested cells were resuspended and sonicated in binding buffer (100 mM Tris–HCl pH 8.0, 500 mM NaCl and 10 mM imidazole) for his-tagged proteins or in GST-A buffer (3.78 mM NaH_2_PO_4_, 16 mM Na_2_HPO_4_ and 150 mM NaCl, pH 7.4) for GST-tagged proteins. The lysate was centrifuged and the supernatant was loaded on the affinity column (his-tagged proteins on Ni-NTA agarose affinity matrix, GST-tagged proteins on Glutathione agarose affinity matrix). The column-bound protein was washed with a wash buffer (100 mM Tris–HCl pH 8.0, 500 mM NaCl and 40 mM imidazole) for his-tagged proteins. GST-tagged proteins were washed with GST-A buffer. The protein was then eluted using an elution buffer (100 mM Tris–HCl pH 8.0, 500 mM NaCl and 250 mM imidazole) for his-tagged proteins. And GST-tagged proteins were eluted with GST-B buffer (3.78 mM NaH_2_PO_4_, 16 mM Na_2_HPO_4_, 150 mM NaCl, and 20 mM L-Glutathione (reduced), pH 7.4) The elution was dialyzed overnight and stored in 20 mM Tris-HCl(pH 7.5), 100 mM NaCl, 10% glycerol, at −20°C. Both 6× his tagged and GST-fused recombinant proteins were prepared for activity and protein–protein interaction assays. Protein concentration was detected by Coomassie Brilliant Blue assay.

### Production of Anti-Ms5082 (MsTAG) and Anti-Ms6939 (MsParA) Antiserums

After immunizations, the rabbit antiserum was collected as previously described [28]. Preimmune serum was collected prior to immunization. Japanese white rabbits were injected with a mixture of 500 µg purified His-tagged MsParA or MsTAG protein mixed with an equal volume of complete Freund’s adjuvant on the back and proximal limbs (100 µl per site). Two weeks later, the rabbits were boosted twice intramuscularly with the same amount of His-tagged MsParA or protein mixed with an equal volume of incomplete Freund’s adjuvant at a two-week interval. 9 days later, the antiserum was harvested from the carotid artery and stored at −80°C for further use.

### Bacterial Two-hybrid Assay

The BacterioMatch II Two-Hybrid System Library Construction Kit (Stratagene) was used to detect protein–protein interactions between ParA and TAG proteins based on transcriptional activation and analysis was carried out according to the manufacturer’s instructions and previously published procedures [29], [30]. Positive growth cotransformants were selected on the Selective Screening Medium plate containing 5 mM 3-amino-1,2,4-triazole (3-AT) (Stratagene), 8 µg/ml streptomycin, 15 µg/ml tetracycline, 34 µg/ml chloramphenicol and 50 µg/ml kanamycin. Cotransformants containing pBT-LGF2 and pTRG-Gal11P (Stratagene) were used as positive controls for an expected growth on the Screening Medium. Cotransformants containing empty vector pBT and pTRG were used as negative controls.

### Co-immunoprecipitation Assays

The *in vivo* interactions between *Tag* and *parA* were analyzed by co-immunoprecipitation (co-IP) assays according to previously published procedures with some modifications [31]. Exponentially growing cells of *M. smegmatis* (1 L) containing the recombinant plasmid pMV261-MsTAG, derived from pMV261 [32], were fixed with 1% formaldehyde for 20 min and fixation was stopped with 0.125 M glycine for 5 min. Cross-linked cells were harvested and resuspended in 10 mL TBSTT buffer (20 mM Tris-HCl [pH 7.5], 150 mM NaCl, 0.1% Tween 20, 0.1% Triton X-100). Co-IP was performed by incubating and shaking 1 mL of mycobacterial cell extract with 2 µL of MsParA antiserum or Ms3759 antiserum as a negative control for 1 h at 4°C. Then, 50 µL of protein A Sepharose was added, and incubation was continued for another hour. The beads were then washed 3 times with 1 mL of the same buffer and centrifuged at 800 g for 1 min. Finally, the beads were resuspended in SDS–PAGE sample buffer. After boiling, the samples were analyzed by western blotting using anti-MsTAG antibody.

### Construction of the MsParA Deletion Mutant of *M. smegmatis* mc^2^155 and Southern Blot Analysis

Knockout of the MsParA gene from *M. smegmatis* mc^2^155 was performed as described previously published procedures with some modifications [33]. A pMind derived suicide plasmid was constructed and a *sacB* gene was inserted to confer sensitivity to sucrose as a negative selection marker. A reporter gene *lacZ* was cloned as another selection marker. The recombinant plasmid pMindMsParA was electrophorated into *M. smegmatis* mc^2^155 and selected on 7H10 medium containing 50 µg/ml hygromycin, 4% sucrose and 60 µg/ml X-gal. Genomic DNA from allelic-exchange mutants in which the MsParA gene had been deleted was identified by PCR analysis using primers on each side of the MsParA and the hygromycin gene.A 300-bp probe corresponding to the sequence of the MsParA upstream genomic fragment of *M. smegmatis* was obtained by PCR using the primer pair 5-AGGATCG AGAGGTACGCGACCGGGTGGGG-3 and 5-TCCGACC CGACTTGTTCCGTCC CGGTTTGG -3). The PCR product was labeled with digoxigenin dUTP (Roche Applied Science) and was used to detect the size change of the *BstE* II*-*digested genomic fragment of *M. smegmatis* before and after recombination. Total DNA of *M. smegmatis* or *M. smegmatis* MsParA::hyg (Msm-MsParA::hyg) was digested completely using *BstE* II, and the resulting fragments were separated by agarose gel electrophoresis (0.8%), transferred to a nylonmembrane, and hybridized with the 300-bp probe. Southern blotting and DNA hybridization were performed according to the manufacturer’s instructions (Roche Applied Science). The filter was developed and photographed.

### Scanning Electron Microscopy (SEM) Observation

*M. smegmatis* cells prepared for scanning electron microscopy (SEM) observation were grown in 7H9 for 24 hours in the presence of 30 µg/mL kanamycin or 0.012% MMS. Cells were harvested by centrifugation. The bacterial pellets were resuspended and incubated at 4°C for 12 hours in 2.5% glutardialdehyde solution. The cells were washed twice in double distilled water, dehydrated by 10 min treatments in different concentrations of ethanol and kept at −80°C for 2 hours. Samples were critical-point dried, sputter-coated with gold, and observed using a scanning electron microscope (S570; Hitachi, Tokyo, Japan).

### Bacterial Growth Assays

Growth assays of Ms/pMV361 [32], Msm-MsParA::hyg/pMV361 and Msm-MsParA::hyg/pMV361-MsParA were conducted in 7H9-Kan-Tw (7H9 medium supplemented with 0.05% Tween 80, 30 µg/mL kanamycin and 0.2% glycerol) media. Cells were grown at 37°C with aeration for 15 hours and samples were collected every 3 h for OD600 determination and microscopic examination.

### Methyl Methanesulfonate (MMS) Sensitivity Assays

MMS is a DNA alkylating agent which modifies both guanine (to 7-methylguanine) and adenine (to 3-methlyladenine) to cause base mispairing and replication blocks, respectively [34]. An overexpression vector pMV261 was used to analyze the sensitivity of the *Tag* gene or its mutant variant to MMS. Wild type or mutant *Tag* gene was cloned next to the heat shock promoter hsp60 in pMV261 to produce corresponding recombinant plasmids which were then transformed into *M. smegmatis*. The strain containing the empty pMV261 plasmid was used as negative control. Cells were grown at 37°C with aeration in 7H9 media with or without 0.012% MMS. Samples were taken at various time points (0, 3, 6, 12 and 15 h) for CFU determination. All assays were performed three times.

### Construction of MsTAG-GFP and MsParA-DsRed2 Fluorescent Fusion Double Overproduction Strains and Protein Co-localization Assays

MsTAG (*Eco*R I + *Hind* III) and MsParA (*Dra* I + *Kpn* I) genes were amplified by polymerase chain reaction (PCR) from *M. smegmatis* genomic DNA using gene-specific primers with appropriate restriction sites (Suppl Table S1). MsTAG was cloned downstream of the heat shock promoter *hsp60* in pMV261, an *E. coli-M. smegmatis* shuttle vector. GFP (*Hind* III + *Cla* I) coding sequence was cloned downstream of and in frame with MsTAG for expression of MsTAG-GFP fusion proteins. To prevent the GFP tag from affecting the folding of MsTAG proteins, a linker (A-G-A-G-K-L) was added between them. The *hsp60* (*Xba* I+ *Dra* I) promoter was cloned into pMV261MsTAG-GFP recombinant vectors in the opposite direction of MsTAG-GFP, and the MsParA (*Dra* I+ *Kpn* I) gene was cloned downstream of the *hsp60* promoter. Finally, the *Dsred2* sequence [35] expressing a red fluorescent protein was cloned next to MsParA to get expression of MsParA-DsRed2 fusion proteins. A linker (T-G-A-G-A) was placed between MsParA and DsRed2 to prevent possible problems with protein folding. The recombinant plasmid pMV261MsTAG-GFP/MsParA-DsRed2 was electroporated into *M. smegmatis*. The resulting recombinant *M. smegmatis* stains were grown in 7H9-Kan-Tw (7H9 medium supplemented with 0.05% Tween 80, 30 µg/mL kanamycin and 0.2% glycerol) media at 37°C for 2 d, then cultured at 42°C for 2 h to increase the level of protein expression. Next, cells were collected and visualized by bright-field and fluorescence microscopy using a Zeiss Axio Scope A1 microscope with a CoolSnap ES CCD camera and a high-pressure mercury lamp. The MsTAG-GFP fusion proteins were imaged using a GFP filter (Ex455-490/Em515-550) and MsParA-DsRed2 fusion proteins were imaged using a TRITC filter (Ex530-560/Em590-650). Digital images were acquired and analyzed with the Image-Pro Plus software (MediaCybernetics, Bethesda, MD).

### 4′,6′-diamidino-2-phenylindole (DAPI) Staining Assays of *M. smegmatis* Cells

*M. smegmatis* cells Ms/pMV261, Ms/pMV261MsTAG and Ms/pMV261- MsTAG-E46A were cultured at 37°C in 7H9 media with 0.012% MMS, and MsParA-deleted mutant strain was grown in 7H9 media without MMS. Cells were harvested, resuspended in phosphate buffered saline (PBS; 10 mM Na_2_HPO_4_, 2 mM KH_2_PO_4_, 137 mM NaCl, 2.7mM KCl, pH 7.4), and stained with DAPI (1 µg/ml, Roche) for 1 h at 37°C. Then the cells were harvested, washed one time with pBS and resuspended in PBS buffer. The samples were examined by bright-field and fluorescence microscopy using a Zeiss Axio Scope. A1 microscope. The DNA localization was imaged with a standard DAPI filter set (Ex330–385/Em420). Digital images were acquired and analyzed with Image-Pro Plus software.

### Site-directed Mutagenesis by Overlap Extension Polymerase Chain Reaction (PCR)

MsTAG-E46A and MsParA-K78A mutants were produced according to the method described previously [36]. Two DNA fragments having overlapping ends were generated by PCR with complementary oligodeoxyribo-nucleotide (oligo) primers (contain the specific alterations). These fragments were combined in a subsequent ‘fusion’ reaction in which the overlapping ends anneal, allowing the 3′ overlap of each strand to serve as a primer for the 3′ extension of the complementary strand. The resulting fusion product was amplified further by PCR. The recombinant plasmids were verified by DNA sequencing.

### ATPase Activity Assay

ATPase activities of ParA and TAG were assayed as described previously [37]. Reactions were performed in a volume of 50 µL containing 50 mM HEPES, pH 8.0, 1 mM MgCl_2_, 200 µM ATP, 150 nM protein at 37°C for 1.5 h. Reactions were terminated by the addition of 50 µL malachite green reagent (1∶1∶2∶2 ratio of 5.7%. ammonium molybdate (w/v) in 6 N HCl, 2.3% (w/v) polyvinyl alcohol (Sigma), 0.1% (w/v) malachite green (Sigma) and distilled water). The color was allowed to stabilize for 5 min before the absorbance was measured at 630 nm. A calibration curve was constructed using 0–25 µmol inorganic phosphate standards and samples were normalized for acid hydrolysis of ATP by the malachite green reagent.

## Results

### Lack of ParA Inhibits Growth and Leads to Cell Elongation in *M. smegmatis*

**Figure 1:**
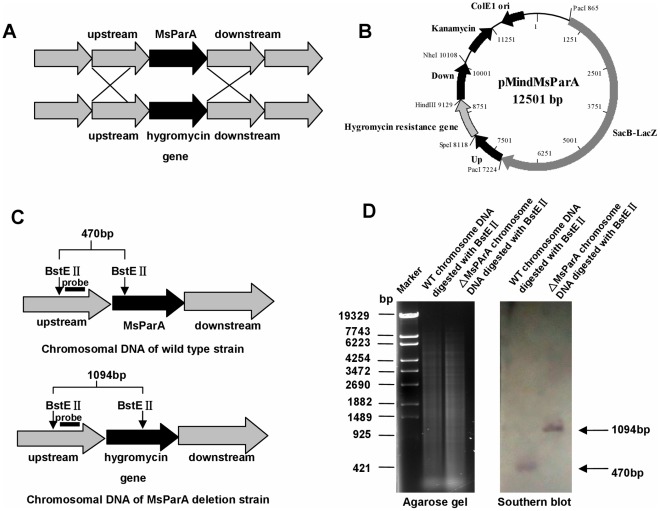
Construction of the MsParA (Ms6939) knockout strain of *M. smegmatis* and Southern blot assays. (**A**) Schematic representation of the recombination strategy for the removal of MsParA from the genome of *M. smegmatis*. (**B**) A map of the recombinant vector pMindMsParA containing upstream and downstream sequences of MsParA, and the gene that confers resistance against hygromycin. (**C**) Schematic representation of the size of a *BstE* II-digested DNA fragment from the genomic DNA of Msm/WT strain (upper panel) and MsParA knockout strain (lower panel). The probe is indicated with a black bar. (**D**) Southern blot assays. A 300 bp probe corresponding to the sequences of the MsParA upstream genomic fragment of *M. smegmatis* was obtained by PCR and labeled with digoxigenin dUTP (Boehringer Mannheim, Inc., Germany). The probe was used to detect the size change of the *BstE* II-digested genomic fragment of *M. smegmatis* before and after recombination.

Previous studies have suggested that either increasing or decreasing ParA expression level in *M. smegmatis* affects bacterial growth [21], [22]. In this study, we first constructed a *parA*-deleted mutant *M. smegmatis* strain to further analyze the effects of ParA on mycobacterial growth and cell morphology. As shown in Figure 1A, an *MsParA*-deleted mutant *M. smegmatis* strain was generated using gene replacement strategy (Fig. 1). A knockout plasmid pMindMsParA containing the Up and Down regions of the MsParA gene was constructed (Fig. 1B). Deletion of MsParA in the mutant strain was further confirmed by a Southern blot assay as shown in Figure 1D. Signal bands of about 1.0 kb and 470 bp were detected in the *Bst*E II-digested genomic DNA of the mutant and wildtype strains (Fig. 1D), respectively, which is consistent with the deletion of MsParA from the chromosomal DNA of *M. smegmatis* in the mutant strain (Fig. 1C).

**Figure 2:**
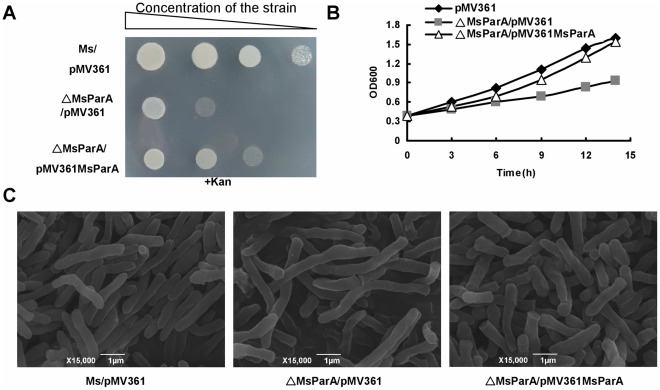
MsParA affects the growth and morphology of *M. smegmatis*. The wild-type and mutant strains were grown on the surface of solid agar medium and in the liquid 7H9 medium. (**A**) Strains were grown on 7H10 agar plates supplemented with 30 µg/ml Kanamycin (Kan) at 37°C for 48 hours. (**B**) Monitoring of growth on 7H9 medium of the *M. smegmatis* wild-type (Ms/pMV361), MsParA deletion strain (Msm-MsParA::hyg/pMV361) and MsParA complementation strain (Msm-MsParA::hyg/pMV361MsParA) by OD600 analysis as described under “Materials and Methods”. (**C**) Scanning electron microscopy assay of cell morphology. The experiment was carried out as described in the “Materials and Methods”. Representative images are shown. The images were taken at 15,000× magnification. Bars, 1 µm.

Next, we measured the growth of mutant and wildtype strains on the surface of solid agar medium and in liquid 7H9 medium. As shown in Figure 2A, when the mycobacterial strains were spotted on the surface of solid agar medium, a thin bacterial lawn was observed for the mutant strain in contrast to the thicker lawn for the wildtype, indicating that the *parA*-deleted mycobacterial strain grew at a slower rate than the wildtype. Expression of *parA* through a pMV361-derived vector could rescue the slow growth phenotype of the mutant strain (Fig. 2A).

We further confirmed the growth difference of the above three strains by determining their growth curves in liquid 7H9 medium. We observed a slower growth rate for the mutant strain while the complement strain, Msm-MsParA::hyg/pMV361-MsParA, grew as well as the wildtype strain (Fig. 2B). Additionally, we found the cell length of the mutant strain to be approximately 2-fold longer at the same time point than that of wildtype *M. smegmatis* cells (Fig. 2C, middle panel). Consistent with the results of our growth experiments, transformation of the mutant strain with a pMV361-derived plasmid containing the *parA* gene (Fig. 2C, right panel) restored the morphology of the mutant strain to wildtype levels.

In summary, the mutant strains lacking *parA* grew slower and their cells were elongated compared to the wildtype. Our rescue experiments indicate that these growth and morphological differences between the two strains can be attributed to the loss of *parA* in the mutant strain.

### ParA Physically Interacts with 3-methyladenine DNA Glycosylase I (TAG) in *M. smegmatis*.

**Figure 3:**
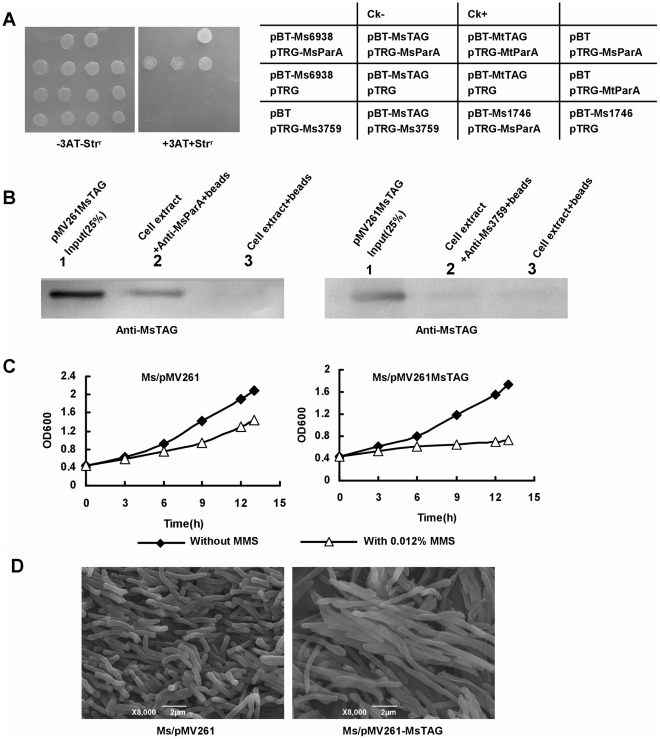
Physical interaction of MsTAG (Ms5082) with MsParA and its effect on mycobacterial growth in response to DNA damage induction. (A) Bacterial two-hybrid assays for the interaction of MsTAG with MsParA performed as described in ‘Materials and Methods’. (B) Co-IP assays. Exponentially growing cells of recombinant *M. smegmatis* containing MsTAG-expression plasmid were harvested, resuspended and lysed. Co-IP assays were performed as described under ‘Materials and Methods’. Right panel shows a negative control using an unrelated anti-Ms3759 anti-serum. (C) MMS sensitivity assays for the MsTAG-overexpressing *M. smegmatis* strains. Growth of the recombinant mycobacterial strains were examined in the presence or absence of 0.012% MMS. Aliquots were taken at the indicated times and the CFU was measured. Each analysis was performed in triplicate. Representative growth curves are shown. The recombinant mycobacterial strains are indicated above the panels. (D) Scanning electron microscopy assay of cell morphology. The recombinant mycobacterial strains were grown in the presence of 0.012% MMS and SEM observation was carried out as described in ‘Materials and Methods’. Representative images are shown. The images were taken at 8000× magnification. Bars, 2 µm.

In a previous global protein-protein interaction analysis [38], the *M. tuberculosis* MtParA, encoded by Rv3918c, was linked to MtTAG, encoded by Rv1210. We assayed the potential physical interaction between their two corresponding *M. smegmatis* homologs–MsParA and MsTAG–to further examine the regulation of ParA. As shown in Figure 3A, in our bacterial two-hybrid assays, the co-transformants containing MsParA and MsTAG grew well on the screening medium. Positive co-transformants (CK^+^) grew on the medium, whereas negative co-transformants (CK^−^) were incapable of growth on the same screening medium. No growth was observed for their self-activated controls, or for the co-transformants of MsParA and a non-specific gene (Ms1746). Consistent with previous results [33], a clear interaction between MtParA and MtTAG was detected (Fig. 3A). These results indicated that MsParA physically interacts with MsTAG in *M. smegmatis.* A further *in vitro* pull-down assay using purified forms of these proteins also confirmed the specific interaction between them (Suppl Fig. S1).

In order to examine the physiological significance of the *in vitro* interactions, we performed co-IP assays for possible *in vivo* interactions between MsParA and MsTAG. Protein A beads that were first conjugated with antibody raised against MsParA were used for the co-IP assay. As shown in Figure 3B, a specific hybridization signal for MsParA in *M. smegmatis* cell extracts (left panel, lane 2) was detected by the anti-MsTAG antibody, albeit at a weaker level than the signal for the positive control MsTAG, which was expressed using a pMV361 plasmid in *M. smegmatis* (Fig. 3B, left panel, lane 1). In contrast, no obvious specific signal was detected for the association in the absence of anti-MsParA antibody in the reactions (lane 3), or in the presence of a non-specific anti-Ms3759 antibody (Fig. 3B, right panel, lane 1).

These results indicate that MsParA can specifically interact with MsTAG both *in vitro* and *in vivo*.

### Overexpression of MsTAG Results in Mycobacterial Growth Inhibition and Cell Elongation Under DNA Damage Stress

In the above assays, MsParA was shown to affect cell growth and morphology, and to interact with MsTAG. This suggested an interesting possibility that MsTAG, which is known to encode a DNA glycosylase, could also be involved in the regulation of mycobacterial morphology. To test this hypothesis, we determined the effects of overexpression of MsTAG on mycobacterial growth. As shown in Figure 3C, overexpression of MsTAG using a pMV361-derived plasmid in *M. smegmatis* (middle panel) caused significant growth inhibition compared to the wildtype strain. The amount of *M. smegmatis* recombinant cells overexpressing MsTAG barely increased after 14 hours under the induction of 0.012% MMS, a DNA damage agent (Fig. 3C, right panel). Furthermore, cell lengths of the MsTAG-overexpressed strains were also observed to be substantially increased (about 4–6 fold) compared to those of wildtype strains (Fig. 3D, right panel). Wildtype and the recombinant strains had no obvious difference in growth (Fig. 3C, left panel) and morphology (Fig. 3D, left panel) in the absence of DNA damage induction.

Thus, overexpression of MsTAG caused growth inhibition and cell elongation of *M. smegmatis* under conditions of DNA damage stress, which is similar to the phenotype of the MsParA-deleted strain.

### The Effect of MsTAG on Mycobacterial Growth is Independent of its DNA Glycosylase Activity

**Figure 4:**
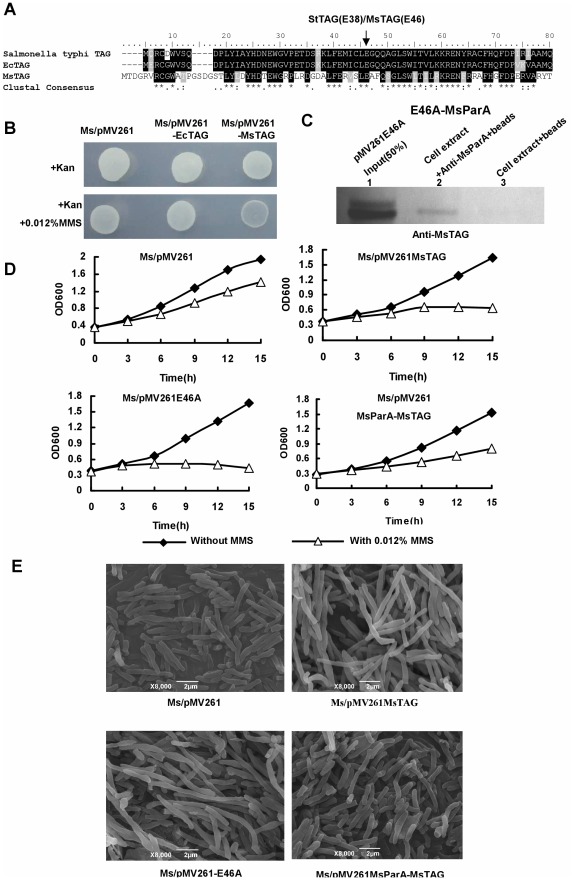
Effects of MsTAG and its co-expression with MsParA on mycobacterial growth and morphology. (**A**) A portion of an alignment of 3-methyladenine DNA glycosylase is shown with conserved catalytic residues Glu (E) indicated by an arrow. (**B**) Comparative growths of *E. coli* overexpressing the *Tag* gene b3459 and *M. smegmatis* strain overexpressing MsTAG on 7H10 agar plates with or without 0.012% MMS at 37°C. (**C**) Co-IP assays for the interaction between the MsTAG-E46A mutant and MsParA. (**D**) MMS sensitivity assays. Growth of *M. smegmatis* strains overexpressing MsTAG or its mutant variant (E46A) and those co-expressing MsTAG and MsParA in 7H9 medium with and without 0.012% MMS were compared. Aliquots were taken at the indicated times and the OD600 was measured as described in ‘Materials and Methods’. Each analysis was performed in triplicate. Representative growth curves are shown. (**E**) Scanning electron microscopy assay of cell morphology. The experiment was carried out as described in ‘Materials and Methods’. The recombinant mycobacterial strains were grown in 7H9 medium supplemented with 0.012% MMS. Representative images are shown. The images were taken at 8000× magnification. Bars, 2 µm.

As shown in Figure 4A, the DNA glycosylase sequence is conserved in several bacterial species including *M. tuberculosis* (MtTAG), *M. smegmatis* (MsTAG) and *E. coli* (b3549). We overexpressed the *E. coli* DNA glycosylase (b1535) in *M. smegmatis* and compared its effects with that of MsTAG. As shown in Figure 4B, *E* *. coli* b1535 had no significant effect on mycobacterial growth compared to the wildtype strain. However, overexpressing MsTAG strikingly inhibited myobacterial growth, suggesting that the effects of MsTAG on mycobacterial growth were not due to its DNA glycosylase activity. To test this further, we constructed a mutant, MsTAG-E46A, in which the N-terminal residue in MsTAG that had been previously shown to be essential for its DNA glycosylase activity [37] was mutated. Interestingly, the mutant lacking DNA glycosylase activity showed significant interaction with MsParA in *M. smegmatis* in our co-IP assays, as shown in Figure 4C. Additionally, overexpression of the mutant gene inhibited growth (Fig. 4D) and caused cell elongation (Fig. 4E) under conditions of DNA damage-induced stress.

Taken together, these results show that the effects of MsTAG on mycobacterial growth and morphology are independent of its function as a DNA glycosylase.

### Co-expression of MsParA with MsTAG Rescues the Growth Defect of Strains Overexpressing MsTAG

A likely explanation for the effect of overexpressing MsTAG on mycobacterial growth and morphology is that overexpression of MsTAG inhibited the function of MsParA via their physical interaction. To test this, we examined the phenotype of strains in which both MsParA and MsTAG were overexpressed. As shown in Figures 4D and 4E, co-expressing MsParA with MsTAG in *M. smegmatis* counteracted the inhibition of bacterial growth (Fig. 4D) and rescued the cell elongation defects (Fig. 4E) caused by overexpression of MsTAG alone.

Further, we examine the effects of MsTAG and MsParA on the mycobacterial cell division. Ms/pMV261, Msm-MsParA::hyg, Ms/pMV261MsTAG and Ms/pMV261-MsTAG-E46A were grown under MMS stress condition. By DAPI staining, one or two chromosomal foci per cell for Ms/pMV261was observed (Suppl. Fig. S2). In contrast, Ms/pMV261-MsTAG, Ms/pMV261-MsTAG-E46A and MsParA-deleted mutant cells were found to contain multiple chromosomal loci (5–7 foci) along the length of the cells (Suppl. Fig. S2), indicating that the deletion of MsParA or overexpression of MsTAG or MsTAG-E46A affected the cell division.

These results indicate that MsTAG affects bacterial growth and cell morphology at least in part by regulating MsParA.

### MsTAG Inhibits the ATPase Activity of MsParA

**Figure 5:**
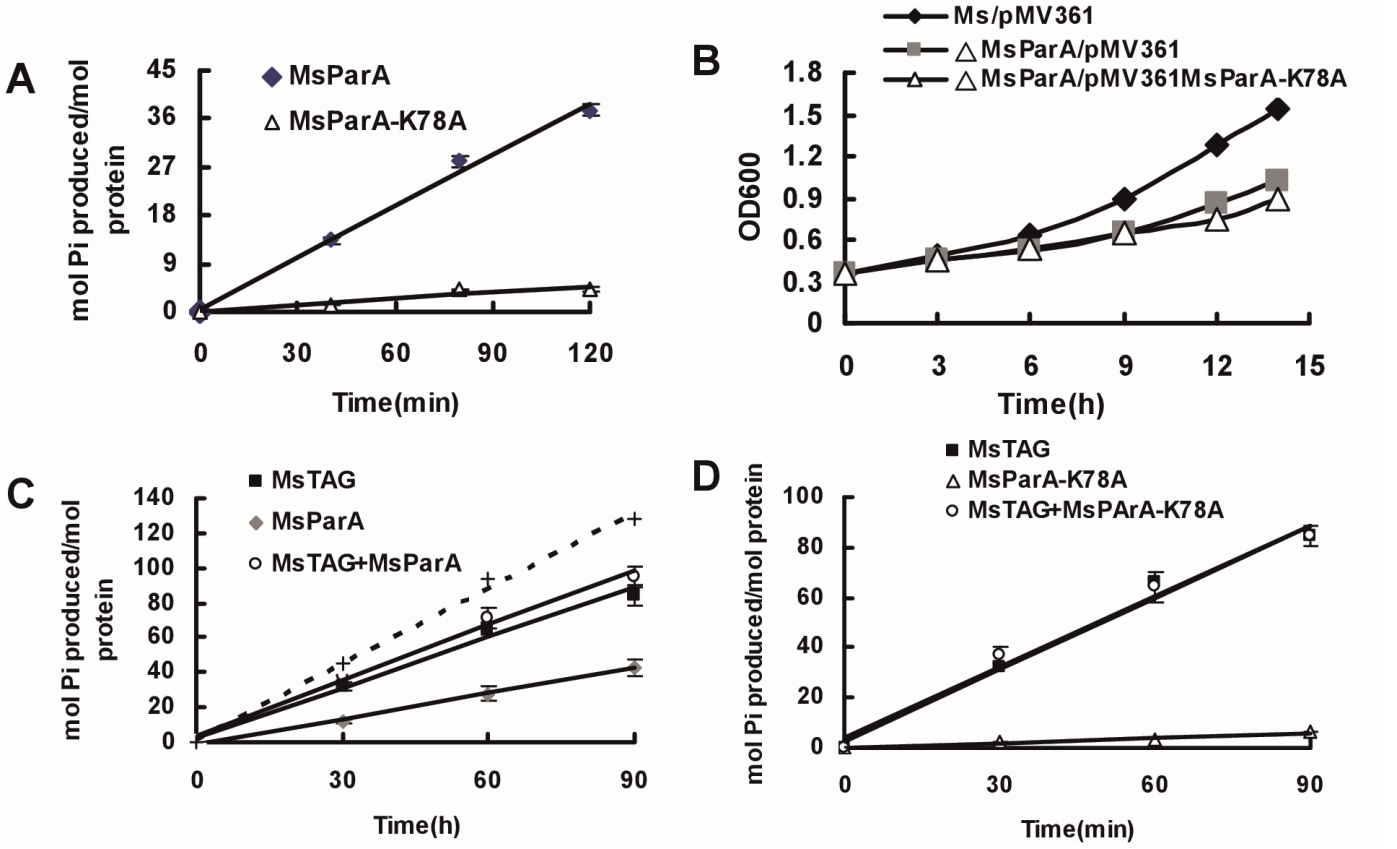
MsTAG regulates the ATPase activity of MsParA. ATPase activity was determined as described under ‘Materials and Methods’. Reactions were performed in a volume of 50 µL and were terminated by the addition of 50 µL malachite green reagent. Absorbance was measured at 630 nm for the color reactions. A calibration curve was constructed using 0–25 µmol inorganic phosphate standards and samples were normalized for acid hydrolysis of ATP by the malachite green reagent. (**A**) Time course ATPase activity assays for ParA and its mutant K78A. (**B**) Monitoring of growth of the *M. smegmatis* wildtype (Ms/pMV361), MsParA deletion strain (Msm-MsParA::hyg/pMV361) and K78A-complementation strain (Msm-MsParA::hyg/pMV361 K78A) in 7H9 medium by CFU analysis as described under ‘Materials and Methods’. (**C**) Effects of MsTAG on MsParA ATPase activity. Equimolar amounts of MsTAG and MsParA were co-incubated at 4°C for 15 min prior to reaction. (**D**) Effects of mutant MsParA (K78A) on MsTAG ATPase activity.

MsParA was previously shown to have ATPase activity, which is required for its role in promoting normal cell division [4]. To further elucidate the regulation of MsParA by MsTAG, we decided to investigate the effect of MsTAG on the ATPase activity of MsParA. Using a color reaction method [18], we found that the ATPase activity of MsParA increased with the addition of increasing amounts of MsParA proteins into the reactions, verifying that MsParA had ATPase activity (Fig. 5A). In contrast, MsParA-K78A, a mutant variant of MsParA in which a residue essential for the activity was mutated [37], [39], exhibited no ATPase activity under similar conditions (Fig. 5A). Interestingly, the mutant also lacked the ability to rescue the growth defects observed in MsParA-deleted mutant strains (Fig. 5B).

Next, we examined whether MsTAG also had ATPase activity and its effect on the activity of MsParA. Curiously, MsTAG was found to have stronger ATPase activity than MsParA under the same conditions (Fig. 5C, left panel). However, when the two proteins were mixed together in a reaction, the activity of the mixture was only close to that of MsTAG alone (Fig. 5C, left panel, circle line) and obviously lower than the expected activity level of MsTAG and MsParA combined (Fig. 5C, left panel, empty line). This strongly suggested that one of the two proteins inhibited the ATPase activity of the other. Further, MsParA could not inhibit the activity of MsTAG when mutant MsParA-K78A lacking ATPase activity was used to evaluate the effect of MsParA on the MsTAG (Fig. 5C, right panel). Taken together, these results indicate that MsTAG inhibits the ATPase activity of MsParA.

### MsTAG Co-localizes with MsParA in *M. smegmatis in vivo*

**Figure 6:**
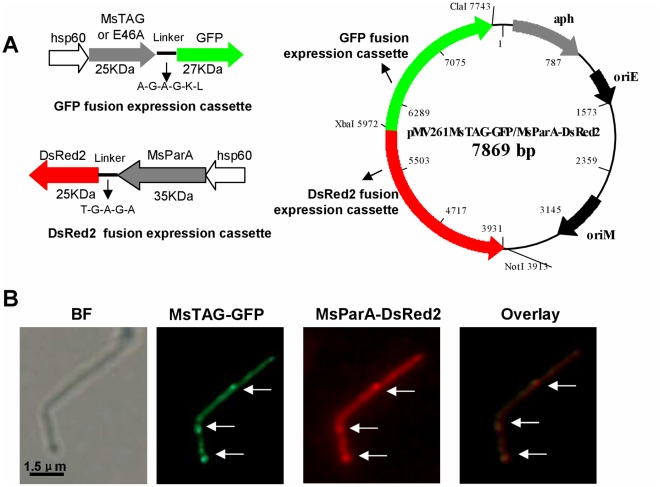
Co-localization assays for MsTAG with MsParA . (**A**) Schematic representation of construction of co-expression plasmids. MsTAG and MsParA were co-expressed under their respective *hsp60* promoters in *M. smegmatis* (left panel). The GFP fusion expression cassette for expressing GFP-fused MsTAG (left upper panel) and the DsRed2 fusion expression cassette for expressing DsRed2-fused MsParA (left lower panel) were constructed as described in ‘Materials and Methods’. The recombinant plasmid pMV261-MsTAG-GFP/MsParA-DsRed2 contained two gene expression cassettes (right panel). (**B**) MsTAG co-localizes with MsParA. The *M. smegmatis* double overexpression strain was grown in 7H9 medium to the stage of logarithmic growth. The localization of MsTAG-GFP and MsParA-DsRed2 within single cells (indicated by arrows) was done by fluorescence microscopy. Images of MsTAG-GFP and MsParA-DsRed2 were further subjected to overlay assay. Yellow fluorescence was observed at points where GFP and DsRed2 signals overlapped, indicating co-localization of the two proteins (right panel).

Since our data indicated physical and functional interactions between MsTAG and MsParA, we predicted that the two proteins would co-localize *in vivo* in *M. smegmatis*. To test this hypothesis, we performed co-localization assays using fluorescently labeled proteins. A recombinant plasmid pMV261-MsTAG-GFP/MsParA-DsRed2 for expressing GFP-fused MsTAG (Fig. 6A, left upper panel) and DsRed2-fused MsParA under individual *hsp60* promoters (Fig. 6A, left lower panel) was designed, constructed and used to make recombinant *M. smegmatis* strains as described in ‘Materials and Methods’. The fusion proteins were clearly expressed in *M. smegmatis* at 42°C, and their characteristic green or red fluorescence could be observed by fluorescence microscopy (Fig. 6B).

We observed that MsTAG and MsParA had similar localization (Fig. 6B). Furthermore, clear yellow fluoresecence could be observed at sites where MsTAG-GFP and MsParA-Red2 signal overlapped, indicating that these two proteins co-localized. There 100 bacterial cells analyzed and co-localization of both proteins is representative for 71.4% of the cases. These results are consistent with our other results indicating physical and functional interaction between these two proteins.

### The Interaction Between TAG and ParA are Conserved in Two Mycobacterial Species

**Figure 7:**
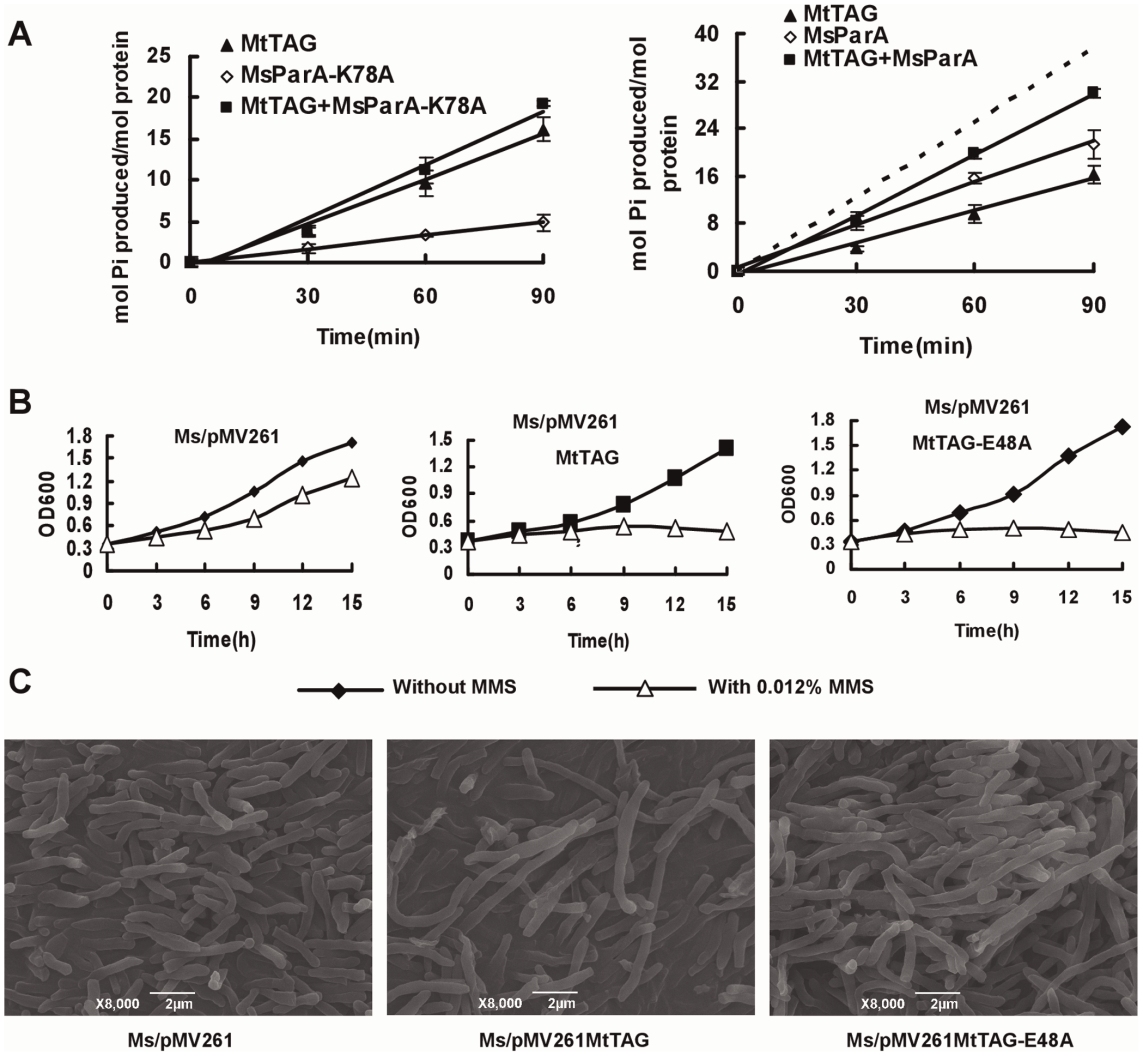
The *M. tuberculosis* MtTAG interacts with MsParA and affects the growth of *M. smegmatis*. (**A**) Effects of MtTAG on the ATPase activity of MsParA. (**B**) Effects of MtTAG and its mutant variants on the growth of *M. smegmatis*. Growth patterns of *M. smegmatis* strains overexpressing MtTAG and its mutant variant (E48A) in the presence of MMS were determined as described under ‘Materials and Methods’. (**C**) Scanning electron microscopy assay of cell morphology. The cells were grown in 7H9 media supplemented with 0.012% MMS and SEM observation was carried out as described in ‘Materials and Methods’. Representative images taken at 8000× magnification are shown.

The ortholog of *M. smegmatis* MsTAG in *M. tuberculosis* is Rv1210 (MtTAG). In the above assays, we had shown that MtTAG interacted with MtParA (Rv3918c). Here we used a co-IP assay and further confirmed the cross-species interaction between the *M. smegmatis* MsParA and MtTAG, which was expressed using a pMind recombinant plasmid in *M. smegmatis*. As shown in Suppl Fig. S3, a specific hybridization signal was detected for MtTAG in *M. smegmatis* cell extracts that were first conjugated with antibody raised against MsTAG. Interestingly, no such signal could be detected for a mutant variant of MtTAG that contained the same mutation (E48A) that disrupted DNA glycosylase in MsParA and was expressed in *M. smegmatis* in a similar manner (right panel). This result indicated to us that *M. tuberculosis* MtTAG might cross-interact with MsParA. Further confirmation of the interaction was obtained by conducting an ATPase activity assay. As shown in Figure 7A, MtTAG had an obvious ATPase activity but Rv1210-K78A, its mutant variant, did not. In addition, MtTAG also exhibited similar inhibition as MsTAG on the ATPase activity of MsParA.

Furthermore, overexpression of MtTAG (Fig. 7B, middle panel) and its mutant form lacking DNA glycosylase activity (Fig. 7B, right panel) in *M. smegmatis* both caused inhibition of growth and substantial increase (4–6 fold) in cell length in the presence of 0.012% MMS compared to the wildtype strain (Fig. 7C).

Taken together, our results show that *M. tuberculosis* MtTAG can cross-interact with *M. smegmatis* MsTAG and inhibit its ATPase activity. Moreover, overexpression of MtTAG had a similar effect as MsTAG on the growth rate and cell morphology of *M. smegmatis*.

## Discussion

ParAB proteins play essential roles in ensuring accurate chromosome segregation and normal cell-cycle [4]. In the present study, we uncovered a novel regulatory mechanism of mycobacterial growth and cell morphology involving a chromosome partitioning protein, ParA. Additionally, we characterized a novel function of 3-methylademine DNA glycosylase (TAG) that is independent of its known role in DNA repair. The mycobacterial TAG was found for the first time to regulate bacterial growth and cell division by directly interacting with ParA and inhibiting its ATPase activity. These findings provide important new insights into the regulatory mechanism of cell growth and division in mycobacteria.

In the current study, a MsParA-deleted mutant strain, Msm-MsParA::hyg, was successfully constructed and the mutant strains grew slower and their cells were elongated compared to the wildtype. These characteristics are similar to those described previously for the *parA* antisense expression strain [20]. Further, we show that the wildtype MsParA gene, but not the mutant MsParA protein deficient in ATP binding (MsParA-K78A), could rescue these defects. Our results thus indicate that ATPase activity of ParA is essential for mycobacterial normal growth, which is consistent with the results of a previous study [16].

The *M. tuberculosis* MtParA (Rv3918c) has been linked to MtTAG in a previous global protein-protein interaction analysis [38]. In the current study, we show that *M. smegmatis* ParA (MsParA) can also interact with 3-methylademine DNA glycosylase both *in vitro* and *in vivo*. 3-methylademine DNA glycosylases remove 3-methyladenine from alkylated DNA and are found widely in prokaryotic and eukaryotic organisms [20], [23]–[26]. However, their functions besides those as a DNA damage and repair enzyme are not known. Here, we provide evidence that the mycobacterial TAG can regulate cell growth and morphology in a DNA repair-independent manner. Additionally, we found that it directly interacts with ParA and inhibits its ATPase activity. We further generated a mutant MsTAG-E46A that lacked DNA glycosylase activity but retained the ability to physically interact with MsParA. Most importantly, the recombinant *M. smegmatis* strains overexpressing MsTAG or its mutant E46A were shown hypersensitive to alkylating agent MMS (Fig. 3 and Fig. 4). In contrast, *E. coli* was insensitive to MMS when following induction of MsTAG expression (Suppl. Fig S4), which was strikingly different from the situation in *M. smegmatis*. The insensitivity is most likely because *E. coli* lacks ParA and ParB [40]. Therefore, the TAG protein could interact with ParA and inhibit its function in *M. smegmatis*, but not in *E. coli*. This model was further supported by the observations that bacterial growth and cell morphology defects could be rescued when TAG was co-expressed with ParA and that TAG (MsTAG) co-localized with ParA (MsParA) in *M. smegmatis*.

Under normal conditions (without MMS stress), MsTAG overexpression had a slight effect on the growth and cell morphology of *M. smegmatis*, which is substantially different from the results we observed under MMS-induced stress. Interestingly, co-expression of MsParA along with MsTAG counteracted the negative effect observed when overexpressing MsTAG alone under conditions of DNA damage-induced stress. These results indicate the possibility that the cooperation between MsTAG and MsParA may be DNA damage–dependent. Under normal conditions, MsTAG is mainly involved in DNA repair activity, maintaining mycobacterial genomic integrity. However, when mycobacteria confront a stressful environment, their genomes are damaged severely. The other known function of MsTAG is controlling the rate of cell division by inhibiting the ATPase activity of ParA. This function of MsTAG might play a major role in contributing to the non-replicating state of *M. tuberculosis* in unfavorable environments.

MtTAG in *M. tuberculosis* has 64% identity and 71% similarity to *M. smegmatis* MsTAG. We found that both of them interacted with MsParA. MtTAG had a similar inhibitory action on MsParA ATPase activity *in vitro* as MsTAG. Moreover, as with MsTAG, *M. smegmatis* became hypersensitive to MMS following overexpression of wildtype MtTAG and its mutant form lacking excision activity (E48A). This implies that MtTAG might regulate cell growth by modulating ParA protein activity in *M. tuberculosis*. Therefore, the specific interaction between two homologous proteins then help the pathogen shift to a dormant state and resistant to inhospitable host-cell and antibiotics.

In recent years, widespread appearance of antibiotic resistance in *M. tuberculosis* has heightened the need to identify new anti-TB drug targets. ParA has been known to act as a chromosome partitioning agent responsible for chromosome segregation and cell growth in both *M. tuberculosis* and *M. smegmatis* [4], [21]. Therefore, ParA has been proposed as a potential target for anti-TB inhibitors. A compound targeting the ATPase activity of ParA has been shown to successfully inhibit the growth of *M. tuberculosis* [22]. In the current study, we observed that mycobacterial growth was obviously inhibited in response to DNA damage induction when MsTAG was overexpressed. Furthermore we showed that MsTAG affected bacterial growth and cell morphology by interacting with MsParA and regulating its ATPase activity. In addition, we confirmed that the interaction was conserved in both *M. tuberculosis* and *M. smegmatis*. Our findings lend further support to the idea that ParA could be an effective target for combating drug resistance in M. tuberculosis.

In summary, we show for the first time that MsTAG physically interacts with MsParA both *in vitro* and *in vivo*. Expression of MsTAG under DNA damage conditions caused growth inhibition of *M. smegmatis*, similar to the effect of deleting the *parA* gene. Further, we showed that the inhibitory role of MsTAG is independent of its DNA glycosylase activity, but instead involves inhibiting the ATPase activity of MsParA. Co-expression of MsTAG and MsParA counteracted the phenotypes observed in strains overexpressing MsTAG alone. Interestingly, MsParA and MsTAG were also found to co-localize in the mycobacterial cells. In addition, the interactions between MsParA and MsTAG were found to be conserved in both *M. tuberculosis* and *M. smegmatis*. Our findings thus provide important new insights on the regulatory mechanisms of cell growth and division in mycobacteria.

## Supporting Information

Figure S1 **Pull-down assays for examining the interaction between MsTAG and MsParA.** Equimolar amounts of GST-Ms3759 or GST-MsParA proteins were combined with equimolar amounts of His-tagged MsTAG proteins in 1.5-ml tubes containing 800 µl of binding buffer (20 mM Tris-HCl, 500 mM NaCl, 5 mM imidazole, pH 7.9). The protein mixtures were gently mixed at 4°C for 2 h. Before further purification, 60 µl of each mixture was removed and saved as a loading control. The remaining mixtures were then incubated with Ni-NTA (Ni^2+^nitrilotriacetate) agarose for another hour and the equimolar amounts of GST-MsParA incubated with Ni-NTA agarose as another negative control. The beads were harvested at 800 g for 1 min and washed with the same buffer for 3 times. The complexs were then eluted with 100 µl elution buffer (20 mM Tris-HCl, 500 mM NaCl, 250 mM imidazole, pH 7.9). The elution was subjected to the SDS-PAGE assay. The protein bands were transferred to a nitrocellulose membrane. Western blot analysis was conducted using primary anti-GST antibody (1∶5,000) and secondary IgG-horseradish peroxidase (goat anti-rabbit) antibody (1∶2,000). The signal was developed using diaminobenizidine (DAB) detection reagents, and the blot was photographed.
(DOC)

Figure S2 **DAPI staining of** ***M. smegmatis*** **cells.** *M. smegmatis* cells Ms/pMV261, Ms/pMV261MsTAG and Ms/pMV261E46A were cultured at 37°C in 7H9 media with 0.012% MMS. While △MsParA strain was grown in 7H9 media without MMS. Cells were harvested, resuspended in phosphate buffered saline (pBS; 10 mM Na_2_HPO_4_, 2mM KH_2_PO_4_, 137 mM NaCl, 2.7mM KCl, pH 7.4), and stained with DAPI(1 µg/ml, Roche) for 1 h at 37°C. Then the cells were harvested, washed one time with pBS and resuspended in pBS. The samples were examined by bright-field and fluorescence microscopy using a Zeiss Axio Scope.A1 microscope. The DNA localization was imaged with a standard DAPI filter set (Ex330–385/Em420). Digital images were acquired and analyzed with Image-Pro Plus software.
(DOC)

Figure S3 **Co-IP assays for the interactions between Rv1210 or its mutant E48A and MsParA** ***in vivo*** **.** Exponentially growing cells of the recombinant *M. smegmatis* containing Rv1210-, or E48A-expression plasmid were harvested, resuspended and lysed. Co-IPs were performed as described under “Materials and Methods”.
(DOC)

Figure S4 **MMS sensitivity of MsTAG overexpression** ***E. coli*** **strain.** Monitoring of sensitivity to MMS of wild-type *E.coli* BL21(DE3) strain [BL21(DE3)/pET28a], MsTAG overexpression strain[BL21(DE3)/pET28a-MsTAG] by optical density analysis. The growth of these recombinant *E. coli* strains were grown at 37°C with aeration in the LB media supplemented with or without 0.012% MMS. Expression was induced by the addition of 0.5 mM IPTG. Samples were taken at various time points (0, 2, 4, 6 and 8 h) for optical density determination. All assays were performed three times. Representative growth curves are shown
(DOC)

Table S1 **Sequences of PCR primers used in this work.**
(DOC)

Table S2 **Plasmids used in this work.**
(DOC)
