# Supplementary material for: Characterization of a Conserved Interaction between DNA Glycosylase and ParA in Mycobacterium smegmatis and M. tuberculosis
Source: PLoS One. 2012 Jun 4;7(6):e38276. doi: 10.1371/journal.pone.0038276 (PMC3366916; doi:10.1371/journal.pone.0038276)
Supplement: Figure S1 — Pull-down assays for examining the interaction between MsTAG and MsParA. Equimolar amounts of GST-Ms3759 or GST-MsParA proteins were combined with equimolar amounts of His-tagged MsTAG proteins in 1.5-ml tubes containing 800 µl of binding buffer (20 mM Tris-HCl, 500 mM NaCl, 5 mM imidazole, pH 7.9). The protein mixtures were gently mixed at 4°C for 2 h. Before further purification, 60 µl of each mixture was removed and saved as a loading control. The remaining mixtures were then incubated with Ni-NTA (Ni2+nitrilotriacetate) agarose for another hour and the equimolar amounts of GST-MsParA incubated with Ni-NTA agarose as another negative control. The beads were harvested at 800 g for 1 min and washed with the same buffer for 3 times. The complexs were then eluted with 100 µl elution buffer (20 mM Tris-HCl, 500 mM NaCl, 250 mM imidazole, pH 7.9). The elution was subjected to the SDS-PAGE assay. The protein bands were transferred to a nitrocellulose membrane. Western blot analysis was conducted using primary anti-GST antibody (1∶5,000) and secondary IgG-horseradish peroxidase (goat anti-rabbit) antibody (1∶2,000). The signal was developed using diaminobenizidine (DAB) detection reagents, and the blot was photographed. (DOC) [file pone.0038276.s001.doc]

**Figure S1**

**FigureS1. Pull-down assays for examining the interaction between MsTAG and MsParA.** Equimolar amounts of GST-Ms3759 or GST-MsParA proteins were combined with equimolar amounts of His-tagged MsTAG proteins in 1.5-ml tubes containing 800 μl of binding buffer (20 mM Tris-HCl, 500 mM NaCl, 5 mM imidazole, pH7.9). The protein mixtures were gently mixed at 4°C for 2h. Before further puriﬁcation, 60 μl of each mixture was removed and saved as a loading control. The remaining mixtures were then incubated with Ni-NTA (Ni^2+^nitrilotriacetate) agarose for another hour and the equimolar amounts of GST-MsParA incubated with Ni-NTA agarose as another negative control. The beads were harvested at 800g for 1 min and washed with the same buffer for 3 times. The complexs were then eluted with 100 μl elution buffer (20 mM Tris-HCl, 500 mM NaCl, 250 mM imidazole, pH7.9). The elution was subjected to the SDS-PAGE assay. The protein bands were transferred to a nitrocellulose membrane. Western blot analysis was conducted using primary anti-GST antibody (1:5,000) and secondary IgG-horseradish peroxidase (goat anti-rabbit) antibody (1:2,000). The signal was developed using diaminobenizidine (DAB) detection reagents, and the blot was photographed.
