# Supplementary material for: Characterization of a Conserved Interaction between DNA Glycosylase and ParA in Mycobacterium smegmatis and M. tuberculosis
Source: PLoS One. 2012 Jun 4;7(6):e38276. doi: 10.1371/journal.pone.0038276 (PMC3366916; doi:10.1371/journal.pone.0038276)
Supplement: Figure S2 — DAPI staining of M. smegmatis cells. M. smegmatis cells Ms/pMV261, Ms/pMV261MsTAG and Ms/pMV261E46A were cultured at 37°C in 7H9 media with 0.012% MMS. While △MsParA strain was grown in 7H9 media without MMS. Cells were harvested, resuspended in phosphate buffered saline (pBS; 10 mM Na2HPO4, 2mM KH2PO4, 137 mM NaCl, 2.7mM KCl, pH 7.4), and stained with DAPI(1 µg/ml, Roche) for 1 h at 37°C. Then the cells were harvested, washed one time with pBS and resuspended in pBS. The samples were examined by bright-field and fluorescence microscopy using a Zeiss Axio Scope.A1 microscope. The DNA localization was imaged with a standard DAPI filter set (Ex330–385/Em420). Digital images were acquired and analyzed with Image-Pro Plus software. (DOC) [file pone.0038276.s002.doc]

**Figure S2**

**Fig. S2 DAPI staining of *M. smegmatis* cells**. *M.smegmatis* cells Ms/pMV261, Ms/pMV261MsTAG and Ms/pMV261E46A were cultured at 37℃ in 7H9 media with 0.012% MMS. While △MsParA strain was grown in 7H9 media without MMS. Cells were harvested, resuspended in phosphate buffered saline (pBS; 10 mM Na_2_HPO_4_, 2mM KH_2_PO_4_, 137 mM NaCl, 2.7mM KCl, pH 7.4), and stained with DAPI(1μg/ml, Roche) for 1h at 37℃. Then the cells were harvested, washed one time with pBS and resuspended in pBS. The samples were examined by bright-ﬁeld and ﬂuorescence microscopy using a Zeiss Axio Scope.A1 microscope. The DNA localization was imaged with a standard DAPI ﬁlter set (Ex330–385/Em420). Digital images were acquired and analyzed with Image-Pro Plus software.
