# Supplementary material for: Characterization of a Conserved Interaction between DNA Glycosylase and ParA in Mycobacterium smegmatis and M. tuberculosis
Source: PLoS One. 2012 Jun 4;7(6):e38276. doi: 10.1371/journal.pone.0038276 (PMC3366916; doi:10.1371/journal.pone.0038276)
Supplement: Figure S3 — Co-IP assays for the interactions between Rv1210 or its mutant E48A and MsParA in vivo . Exponentially growing cells of the recombinant M. smegmatis containing Rv1210-, or E48A-expression plasmid were harvested, resuspended and lysed. Co-IPs were performed as described under “Materials and Methods”. (DOC) [file pone.0038276.s003.doc]

**Figure S3**

**Figure S3. Co-IP assays for the interactions between Rv1210 or its mutant E48A and MsParA *in vivo*.** Exponentially growing cells of the recombinant *M. smegmatis* containing Rv1210-, or E48A-expression plasmid were harvested, resuspended and lysed. Co-IPs were performed as described under “Materials and Methods”.
