# Supplementary material for: Characterization of a Conserved Interaction between DNA Glycosylase and ParA in Mycobacterium smegmatis and M. tuberculosis
Source: PLoS One. 2012 Jun 4;7(6):e38276. doi: 10.1371/journal.pone.0038276 (PMC3366916; doi:10.1371/journal.pone.0038276)
Supplement: Figure S4 — MMS sensitivity of MsTAG overexpression E. coli strain. Monitoring of sensitivity to MMS of wild-type E.coli BL21(DE3) strain [BL21(DE3)/pET28a], MsTAG overexpression strain[BL21(DE3)/pET28a-MsTAG] by optical density analysis. The growth of these recombinant E. coli strains were grown at 37°C with aeration in the LB media supplemented with or without 0.012% MMS. Expression was induced by the addition of 0.5 mM IPTG. Samples were taken at various time points (0, 2, 4, 6 and 8 h) for optical density determination. All assays were performed three times. Representative growth curves are shown (DOC) [file pone.0038276.s004.doc]

**Figure S4**

**BL21(DE3)/pET28a-MsTAG**

**Figure S4. MMS sensitivity of MsTAG overexpression *E. coli* strain.** Monitoring of sensitivity to MMS of wild-type *E.coli* BL21(DE3) strain [BL21(DE3)/pET28a], MsTAG overexpression strain[BL21(DE3)/pET28a-MsTAG] by optical density analysis. The growth of these recombinant *E. coli* strains were grown at 37℃ with aeration in the LB media supplemented with or without 0.012% MMS. Expression was induced by the addition of 0.5mM IPTG. Samples were taken at various time points (0, 2, 4, 6 and 8h) for optical density determination. All assays were performed three times. Representative growth curves are shown.
