# Supplementary material for: Characterization of a Conserved Interaction between DNA Glycosylase and ParA in Mycobacterium smegmatis and M. tuberculosis
Source: PLoS One. 2012 Jun 4;7(6):e38276. doi: 10.1371/journal.pone.0038276 (PMC3366916; doi:10.1371/journal.pone.0038276)
Supplement: Table S1 — Sequences of PCR primers used in this work. (DOC) [file pone.0038276.s005.doc]

**Tables S1**

Table S1 Sequences of PCR primers used in this work

| Name | Sequence 5’→3’ | Enzyme | Usage |
| --- | --- | --- | --- |
| MsParAup f | AGGCTTAATTAACCGATGACTCCGTTCGAG | *Pac*Ⅰ | Knock out |
| MsParAup r | ATATAACTAGTGCTCATCTCCTGCCCGTTC | *Spe*Ⅰ | Knock out |
| MsParAdn f | AGCGAAGCTTTGCAGCATGATCAGTAGGCG | *Hind*Ⅲ | Knock out |
| MsParAdn r | ATATTGCTAGCTCCTGGAGACCCGGCATCT | *Nhe*Ⅰ | Knock out |
| MsParA f | GCCAGAATTCGCATGGGTTCGGGTCAGA | *Eco*RⅠ | Clone to pMV361/ expression |
| MsParA r | ATATAAGCTTCTACTGCTGGCGCGGCGG | *Hind*Ⅲ | Clone to pMV361 |
| MsParA r | ATATTCTAGACTACTGCTGGCGCGGCGG | *Xba*Ⅰ | Clone and expression |
| MsTAG f | ATAGGAATTCGAGTGACCGACGGACGTGTC | *Eco*RⅠ | Clone and expresson |
| MsTAG r | ATATTCTAGACTATCCGGGGCGCAGGGTGT | *Xba*Ⅰ | Clone and expresson |
| Ms6938 f | AGAGGAATTCGAATGAATCAGCCGGCACG | *Eco*RⅠ | Clone to pBT |
| Ms6938 r | ACACTCTAGATTACTCGTTCTGGGCGCTC | *Xba*Ⅰ | Clone to pBT |
| Rv1210 f | ATATGAATTCGTGTGAGCGGCGACGGGCTGGT | *Eco*RⅠ | Clone to pBT, pMV261, pMV361 |
| Rv1210 r | ATATATTCTAGATCACCGGGCCGCCATCGGGC | *Xba*Ⅰ | Clone to pBT, pMV261 |
| Rv1210 r | TATATAAGCTTTCACCGGGCCGCCATCGGG | *Hind*Ⅲ | Clone to pMV361 |
| Rv3918c f | ATTTTATGCGGCCGCAGTGAGTGCTCCGTGGG | *Eco*RⅠ | Clone to pBT |
| Rv3918c r | GCCACGCACCTCTAGATCATGGTCGTCCCTTC | *Xba*Ⅰ | Clone to pBT |
| b3459 f | ATATGAATTCTTATGGAACGTTGCGGCTGG | *Eco*RⅠ | Clone to pMV261 |
| b3459 r | GCGTTCTAGATCATGGTTTATTTCCCGGAT | *Xba*Ⅰ | Clone to pMV261 |
| E46A-1 f | GAGCGAATTCGCGTGACCGACGGACGTGTCC | *Eco*RⅠ | Point mutation |
| E46A-1 r | TCTGGAACGCTGCGAGGCTCACGCGCTCGAA |  | Point mutation |
| E46A-2 f | TTCGAGCGCGTGAGCCTCGCAGCGTTCCAGA |  | Point mutation |
| E46A-2 r | GCGATCTAGACTATCCGGGGCGCAGGGTGTG | *Xba*Ⅰ | Point mutation |
| MsTAG f | ATATTCTAGAGTGACCGACGGACGTGTCCG | *Xba*Ⅰ | Co-expression with MsParA |
| MsTAG r | ATATAAGCTTCTATCCGGGGCGCAGGGTGT | *Hind*Ⅲ | Co-expression with MsParA |
| MsTAG r | ATATAAGCTTTCCGGGGCGCAGGGTGTGCG  (without TAG) | *Hind*Ⅲ | Fusion expression with GFP |
| groELp f | ATATATTCTAGAGGTGACCACAACGACGCGCC | *Xba*Ⅰ | Colocalization |
| groELp r | GCGCGCTTTAAACGCAATTGTCTTGGCCATTG | *Dra*Ⅰ | Colocalization |
| MsParA f | GCGCGCGGCTTTAAAATGGGTTCGGGTCAGAACAAAGGACAGGGC | *Dra*Ⅰ | Colocalization |
| MsParA r | ATTTATATAATAGGTACCAGCACCTGCCTGCTGGCGCGGCGGCGC | *Kpn*Ⅰ | Colocalization |
| RFP f | GCGCGAGGTACCATGGCCTCCTCCGAGAACGT | *Kpn*Ⅰ | Colocalization |
| RFP r | ATAGCGGCCGCCTACAGGAACAGGTGGTGGCG | *Not*Ⅰ | Colocalization |
| K78A-1 f | GGGCCCGAATTCGCATGGGTTCGGGTCAGAAC  AAAGG | *Eco*RⅠ | Point mutation |
| K78A-1 r | TTGACTGCCGTCGTCGTTGCGCCGACGCCGCC  CTT |  | Point mutation |
| K78A-2 f | AAGGGCGGCGTCGGCGCAACGACGACGGCAG  TCAA |  | Point mutation |
| K78A-2 r | ACACTCTAGACTACTGCTGGCGCGGCGGCGCC  CCA | *Xba*Ⅰ | Point mutation |
| E48A-1 f | ATATGAATTCGTGTGAGCGGCGACGGGCTGGT | *Eco*RⅠ | Point mutation |
| E48A-1 r | CACTCTGGAAGGCTGCCAGGCTCATTCGCT |  | Point mutation |
| E48A-2 f | AGCGAATGAGCCTGGCAGCCTTCCAGAGTG |  | Point mutation |
| E48A-2 r | ATATATTCTAGATCACCGGGCCGCCATCGGGC | *Xba*Ⅰ | Point mutation |
| MsTAG up f | GCGTTAATTAAGTCCCGAAGCTGCTGGGAC | *Pac*Ⅰ | Knock out |
| MsTAG up r | ATATAACTAGTGACGTGCCTTCCTCCCCGC | *Spe*Ⅰ | Knock out |
| MsTAG dn f | GCGCAAGCTTGGAACAATAGGGGTTAGTTC | *Hind*Ⅲ | Knock out |
| MsTAG dn r | ATATGCTAGCATCGACACCACGGTCTGGTA | *Nhe*Ⅰ | Knock out |
