# Supplementary material for: Characterization of a Conserved Interaction between DNA Glycosylase and ParA in Mycobacterium smegmatis and M. tuberculosis
Source: PLoS One. 2012 Jun 4;7(6):e38276. doi: 10.1371/journal.pone.0038276 (PMC3366916; doi:10.1371/journal.pone.0038276)
Supplement: Table S2 — Plasmids used in this work. (DOC) [file pone.0038276.s006.doc]

**Tables S2**

Table S2. Plasmids used in this work

| Plasmid Relevant features Source | | |
| --- | --- | --- |
| pMind-MsParA | MsParA gene upstream 890bp and downstream 973bp cloned in pMind | This work |
| pMV361-MsParA | MsParA gene cloned in pMV361 | This work |
| pBT-MsTAG | MsTAG gene cloned in pBT | This work |
| pTRG-MsParA | MsParA gene cloned in pTRG | This work |
| pBT-Ms6938 | Ms6938 gene cloned in pBT | This work |
| pBT-Ms1746 | Ms1746 gene cloned in pBT | This work |
| pBT-Rv1210 | Rv1210 gene cloned in pBT | This work |
| pTRG-Rv3918c | Rv3918c gene cloned in pTRG | This work |
| pTRG-Ms3759 | Ms3759 gene cloned in pTRG | This work |
| pMV261-b3459 | b3459 gene cloned in pMV261 | This work |
| pMV261-MsTAG | MsTAG gene cloned in pMV261 | This work |
| pMV261-E46A | MsTAG mutant E46A gene cloned in pMV261 | This work |
| pMV261-MsParA-MsTAG | MsTAG and MsParA gene cloned in pMV261 | This work |
| pET-MsParA | MsParA gene cloned in pET | This work |
| pET-K78A | MsParA mutant K78A gene cloned in pET | This work |
| pMV361-K78A | MsParA mutant K78A gene cloned in pMV361 | This work |
| pET-MsTAG | MsTAG gene cloned in pET | This work |
| pMV261-MsTAGGFP | MsTAG and GFP gene cloned in pMV261 | This work |
| pMV261-E46AGFP | E46A and GFP gene cloned in pMV261 | This work |
| pMV261-MsTAGGFP-MsParADsRed | MsParA and DsRed gene cloned in pMV261MsTAGGFP | This work |
| pMV261-E46AGFP-MsParADsRed | MsParA and DsRed gene cloned in pMV261E46AGFP | This work |
| pET-Rv1210 | Rv1210 gene cloned in pET | This work |
| pMV261-Rv1210 | Rv1210 gene cloned in pMV261 | This work |
| pMV261-E48A | Rv1210 mutant E48A gene cloned in pMV261 | This work |
| pMind-MsTAG | MsTAG gene upstream 944bp and downstream 986bp cloned in pMind | This work |
| pMV361-MsTAG | MsTAG gene cloned in pMV361 | This work |
| pMV361-E46A | MsTAG mutant E46A gene cloned in pMV361 | This work |
| pMV361-Rv1210 | Rv1210 gene cloned in pMV361 | This work |
| pMV361-E48A | Rv1210 mutant E48A gene cloned in pMV361 | This work |
| pGEX-MsParA | MsParA gene cloned in pET | This work |
| pGEX-Ms3759 | Ms3759 gene cloned in pGEX | This work |
